# Acknowledgments

**DOI:** 10.1002/ags3.70154

**Published:** 2026-01-01

The publication of invaluable papers in *Annals of Gastroenterological Surgery* depends on the prompt, careful review of submitted manuscripts. We would like to thank the following experts for reviewing manuscripts submitted between December 1, 2024 and November 30, 2025.

Abe, Yuta

Aizawa, Masaki

Ajiki, Tetsuo

Akagi, Tomonori

Akamatsu, Nobuhisa

Akita, Hirofumi

Akiyama, Shintaro

Akiyoshi, Takashi

Ando, Koji

Aoki, Takeshi

Aoki, Taku

Aoyagi, Yasuko

Aoyama, Toru

Araki, Kenichiro

Arigami, Takaaki

Arima, Kota

Asai, Koji

Asano, Toshimichi

Baba, Kenji

Bekki, Yuki

Booka, Eisuke

Daiko, Hiroyuki

Ebata, Tomoki

Eguchi, Hidetoshi

Endo, Shungo

Eo, Wankyu

Etoh, Tsuyoshi

Fujio, Atsushi

Fujita, Fumihiko

Fujita, Takeo

Fujiyoshi, Kenji

Fukami, Yasuyuki

Fukui, Yudai

Fukushima, Ryoji

Furuhata, Tomohisa

Furukawa, Kenei

Fuse, Masahiro

Ganeko, Riki

Gohda, Yoshimasa

Goto, Toru

Hagi, Takaomi

Hamabe, Atsushi

Hamada, Madoka

Hanaoka, Marie

Harada, Kazuto

Harimoto, Norifumi

Haruki, Koichiro

Hasegawa, Kiyoshi

Hasegawa, Suguru

Hasegawa, Yasushi

Hashimoto, Daisuke

Hashimoto, Masashi

Hatano, Etsuro

Hayama, Tamuro

Hayami, Shinya

Hayano, Koichi

Hayashi, Hiromitsu

Hayata, Keiji

Hibi, Taizo

Hida, Koya

Hidaka, Masaaki

Hirano, Satoshi

Hirano, Yasumitsu

Hirashita, Teijiro

Hisaka, Toru

Hiyoshi, Yukiharu

Honda, Goro

Honda, Michitaka

Hosoda, Kei

Hu, Qingjiang

Ichikawa, Nobuki

Igaki, Takahiro

Ikenaga, Naoki

Ikeuchi, Hiroki

Ikoma, Hisashi

Imai, Katsunori

Inaki, Noriyuki

Inoue, Mikihiro

Inoue, Yosuke

Irino, Tomoyuki

Ishido, Keinosuke

Ishiyama, Yasuhiro

Ishizawa, Takeaki

Ishizuka, Mitsuru

Ito, Sono

Ito, Takashi

Itoh, Shinji

Iwashita, Yoshiaki

Jiang, Weizhong

Kagawa, Hiroyasu

Kagawa, Yoshinori

Kaibori, Masaki

Kaido, Toshimi

Kajiwara, Yoshiki

Kakisaka, Tatsuhiko

Kakiuchi, Nobuyuki

Kaku, Keizo

Kanaji, Shingo

Kanemitsu, Yukihide

Kanetaka, Kengo

Kasai, Shunsuke

Kato, Yutaro

Kawaguchi, Yoshihiko

Kawai, Kazushige

Kawai, Manabu

Kawaida, Hiromichi

Kawamura, Junichiro

Kawamura, Mikio

Kawanaka, Hirofumi

Kawazoe, Tetsuro

Kikuchi, Hirotoshi

Kimura, Yasutoshi

Kimura, Yutaka

Kinoshita, Takahiro

Kishi, Yoji

Kishino, Takayoshi

Kitago, Minoru

Kitahata, Yuji

Kitami, Chie

Kitano, Shoichi

Kobayashi, Hirotoshi

Kobayashi, Shogo

Kobayashi, Tsuyoshi

Koike, Yuhki

Komatsu, Shohei

Komatsu, Shuhei

Komori, Koji

Konishi, Hirotaka

Kosaka, Hisashi

Kosuga, Toshiyuki

Kosumi, Keisuke

Kouno, Nobuji

Koyama, Fumikazu

Koyanagi, Kazuo

Kubota, Takeshi

Kumazu, Yuta

Kunisaki, Chikara

Kuroda, Shinji

Lee, Sang‐Woong

Makino, Isamu

Makino, Tomoki

Makuuchi, Rie

Manabe, Tatsuya

Maruyama, Suguru

Matsuda, Akihisa

Matsuda, Keiji

Matsuda, Kenji

Matsuhashi, Nobuhisa

Matsui, Ryota

Matsumiya, Yuriko

Matsumoto, Ippei

Matsuo, Yasuko

Matsushima, Hajime

Matsuyama, Ryusei

Matsuyama, Takatoshi

Mayanagi, Shuhei

Mima, Kosuke

Mimura, Toshiki

Mine, Shinji

Misawa, Takeyuki

Mise, Yoshihiro

Mita, Atsuyoshi

Miura, Fumihiko

Miyamoto, Yuji

Miyasaka, Yoshihiro

Miyazaki, Yasuhiro

Miyo, Masaaki

Mizoguchi, Masako

Mizuno, Rei

Mizuno, Ryosuke

Mizuno, Shugo

Mizuno, Takashi

Mori, Shinichiro

Mori, Yoshiko

Morikawa, Takanori

Morimura, Ryo

Morine, Yuji

Morise, Zenichi

Motoi, Fuyuhiko

Motomura, Takashi

Motoyama, Satoru

Murakami, Takashi

Nagai, Minako

Nagakawa, Yuichi

Naitoh, Takeshi

Nakagawa, Kei

Nakajima, Masanobu

Nakamura, Ikuo

Nakamura, Toru

Nakanishi, Yuki

Nakanoko, Tomonori

Nakashima, Yuichiro

Nakata, Kohei

Nakauchi, Masaya

Nakazawa, Nobuhiro

Nanashima, Atsushi

Nanno, Yoshihide

Nara, Satoshi

Ninomiya, Mizuki

Nishidate, Toshihiko

Nishijima, Tomohiro

Nishimura, Atsushi

Nishino, Hitoe

Nishiwada, Satoshi

Nishizawa, Yuji

Nitta, Hiroyuki

Noda, Takehiro

Noji, Takehiro

Noma, Kazuhiro

Noshiro, Hirokazu

Numata, Masakatsu

Nunobe, Souya

Oba, Atsushi

Oba, Takuya

Obama, Kazutaka

Obara, Hideaki

Ochiai, Toshiya

Ogawa, Katsuhiro

Ogino, Takayuki

Ogura, Atsushi

Ohashi, Manabu

Ohtsuka, Masayuki

Ohtsuka, Takao

Okada, Ken‐ichi

Okajima, Hideaki

Okamoto, Kohji

Okamoto, Koichi

Okamoto, Michio

Okamoto, Tatsuya

Okamura, Akihiko

Okamura, Yukiyasu

Okano, Keiichi

Okita, Yoshiki

Okuchi, Yoshihisa

Okugawa, Yoshinaga

Okumura, Shintaro

Okuya, Koichi

Omori, Takeshi

Orimo, Tatsuya

Oshima, Nobu

Otsu, Hajime

Otsuka, Koki

Ouchi, Akira

de Paula, Thais Reif

Ri, Motonari

Saito, Takuro

Saito, Yu

Sakai, Makoto

Sakai, Yoshihiro

Sakamoto, Takashi

Sakamoto, Yoshihiro

Sano, Akihiko

Sasaki, Akira

Sasaki, Ken

Sato, Hiroshi

Sato, Ryuichiro

Sato, Yusuke

Seo, Satoru

Shibao, Kazunori

Shibasaki, Susumu

Shibata, Chikashi

Shibutani, Masatsune

Shichinohe, Toshiaki

Shida, Dai

Shigeno, Takashi

Shigeta, Kohei

Shimada, Yoshifumi

Shimura, Tadanobu

Shindoh, Junichi

Shinoda, Masahiro

Shinto, Eiji

Shiomi, Akio

Shiozaki, Atsushi

Shirai, Yoshihiro

Shiraishi, Takuya

Shoda, Katsutoshi

Soejima, Yuji

Sohda, Makoto

Soyama, Akihiko

Suda, Koichi

Sugawara, Kotaro

Sugimachi, Keishi

Sugiura, Teiichi

Sunami, Eiji

Suto, Hironobu

Suzuki, Shuji

Takahara, Takeshi

Takahashi, Hidekazu

Takahashi, Hidenori

Takahashi, Kenichi

Takahashi, Ryo

Takahashi, Takao

Takahashi, Tsuyoshi

Takahashi, Yusuke

Takami, Hideki

Takamoto, Takeshi

Takashima, Atsuo

Takatsuki, Mitsuhisa

Takayashiki, Tsukasa

Takeda, Mitsunobu

Takeishi, Kazuki

Takeno, Shinsuke

Takeuchi, Masashi

Tanaka, Keitaro

Tanaka, Kimitaka

Tanaka, Kuniya

Tanemura, Masahiro

Tani, Masaji

Taniai, Tomohiko

Taniguchi, Koji

Taniyama, Yusuke

Tazawa, Miyako

Teraishi, Fuminori

Tokunaga, Masanori

Tomimaru, Yoshito

Tominaga, Tetsuro

Toshima, Takeo

Toyozumi, Takeshi

Tsukamoto, Fumio

Tsunoda, Shigeru

Uchida, Yoichiro

Uchino, Motoi

Uemura, Kenichiro

Uemura, Mamoru

Ueno, Masaki

Umeda, Yuzo

Ushigome, Hajime

Wakasugi, Masaki

Wakita, Akiyuki

Watanabe, Manabu

Xiao, Miao

Yagi, Shintaro

Yamada, Suguru

Yamada, Takeshi

Yamaguchi, Tatsuro

Yamaguchi, Tomohiro

Yamakawa, Yushi

Yamamoto, Hirofumi

Yamamoto, Kazuyoshi

Yamamoto, Seiichiro

Yamamoto, Takehito

Yamamoto, Yusuke

Yamashita, Kohei

Yamashita, Kotaro

Yamauchi, Shinichi

Yanagimoto, Yoshitomo

Yano, Fumiaki

Yano, Hideaki

Yasuda, Satoshi

Yasuda, Takushi

Yasufuku, Itaru

Yasui, Masayoshi

Yatabe, Yusuke

Yoh, Tomoaki

Yokoi, Ryoma

Yokoo, Hideki

Yokota, Kazuki

Yoshikawa, Kozo

Yoshikawa, Takaki

Yoshimatsu, Kazuhiko

Yoshimura, Yoshihiro

Yoshitomi, Hideyuki

Yoshiya, Shohei

Yuda, Masami

Yugawa, Kyohei

Yura, Masahiro

